# Mortality and Serious Adverse Events Associated With Glucagon-Like Peptide-1 Receptor Agonists: A Pharmacovigilance Study Using the FDA Adverse Event Reporting System

**DOI:** 10.7759/cureus.65989

**Published:** 2024-08-02

**Authors:** Mehul Bhattacharyya, Larry E Miller, Anna L Miller, Ruemon Bhattacharyya

**Affiliations:** 1 Clinical Research, Miller Scientific, Johnson City, USA; 2 Public Affairs and Economics, University of California Los Angeles, Los Angeles, USA

**Keywords:** safety, obesity, glp-1ra, diabetes, faers, adverse event

## Abstract

Purpose

Glucagon-like peptide-1 receptor agonists (GLP-1RAs) are an increasingly prevalent class of drugs for managing overweight/obesity and type 2 diabetes mellitus. Postmarket surveillance is essential for characterizing their risk profiles in real-world patient populations as clinical use increases. This study investigated the association of GLP-1RAs with mortality and serious adverse events (AEs) reported to the Food and Drug Administration (FDA) Adverse Event Reporting System (FAERS).

Methods

A disproportionality analysis was conducted utilizing FAERS data from Q2 2005 to Q1 2024 to identify AEs listing an approved GLP-1RA as the primary suspect drug. The reporting odds ratio (ROR) was calculated for mortality and serious AEs associated with each GLP-1RA compared to the combined group of all other GLP-1RAs. A signal of disproportionate reporting indicating a potential safety concern was defined as a lower bound of the 95% CI for the ROR exceeding 1.0.

Results

The analysis identified 287,201 AEs associated with GLP-1RAs during the study period. Disproportionality analyses revealed statistically significant elevated signals for mortality with the earliest approved GLP-1RAs: Byetta (ROR = 2.20, 95% CI: 2.06-2.34) and Victoza (ROR = 2.12, 95% CI: 1.98-2.28). Significant elevated signals for serious AEs were identified with the semaglutide products Ozempic (ROR = 2.77, 95% CI: 2.69-2.85), Rybelsus (ROR = 2.42, 95% CI: 2.26-2.60), and Wegovy (ROR = 1.30, 95% CI: 1.22-1.39); the liraglutide products Victoza (ROR = 2.10, 95% CI: 2.04-2.15) and Saxenda (ROR = 2.21, 95% CI: 2.09-2.33); and Byetta (ROR = 1.11, 95% CI: 1.08-1.14) compared to other GLP-1RAs. The newer GLP-1RAs were associated with a higher proportion of serious AEs reported in younger patients (p < 0.001) and females (p < 0.001).

Conclusion

This pharmacovigilance study utilizing the FAERS database identified potential safety signals of increased mortality and serious AE reporting associated with certain GLP-1RAs, particularly the earlier approved liraglutide agents Byetta and Victoza. These findings highlight the importance of proactive postmarket surveillance to characterize the real-world safety profiles of individual GLP-1RA drugs.

## Introduction

Obesity and type 2 diabetes mellitus represent significant and growing public health challenges worldwide. In the United States, the age-adjusted prevalence of obesity was 42% in 2017-2018, reflecting a concerning upward trend over the last two decades [1]. Consequently, the associated burden of this disease is expected to continue rising [2], given the association of obesity with numerous chronic diseases, including cardiovascular disease, osteoarthritis, and certain cancers. Simultaneously, the global prevalence of type 2 diabetes mellitus continues to rise [3], driven by the obesity epidemic, population aging, and other risk factors like physical inactivity and an unhealthy diet.

Lifestyle modifications and bariatric surgery have demonstrated efficacy in treating obesity. However, long-term weight loss maintenance remains a challenge, with an estimated 80% of lost weight regained within five years [4]. Pharmacological options for obesity management have expanded in recent years, yet many anti-obesity agents have limited efficacy, unacceptable adverse events (AEs), or restrictions on treatment duration, thus highlighting the need for novel therapeutic approaches [5]. Similarly, existing antidiabetic medications may improve glycemic control, but their impact on long-term disease progression varies, prompting consideration of additional treatment options.

Glucagon-like peptide-1 receptor agonists (GLP-1RAs) have garnered considerable attention for the management of overweight/obesity and type 2 diabetes mellitus. GLP-1 is an incretin hormone secreted by enteroendocrine L-cells in response to nutrient ingestion that enhances glucose-dependent insulin secretion, suppresses glucagon release, slows gastric emptying, and promotes satiety [6]. GLP-1RAs mimic these actions while exhibiting resistance to degradation by dipeptidyl peptidase-4, resulting in prolonged pharmacological activity [7]. Since the US FDA approved the first GLP-1RA in 2005 (exenatide (Byetta)), this class of drugs has demonstrated efficacy in promoting weight loss [8] and improving glycemic control in diabetic patients [9] in multiple clinical trials. Subsequently, over one million Americans received a GLP-1RA prescription between 2018 and 2023, reflecting their widespread adoption [10].

Despite sharing a common mechanism of action, individual GLP-1RAs exhibit distinct pharmacokinetic and pharmacodynamic properties that may impact their safety and tolerability profiles. For example, semaglutide and dulaglutide have substantially longer half-lives (seven days and five days, respectively) than exenatide (two hours) and lixisenatide (three hours), which influences dosing requirements and the potential for drug accumulation [11]. Semaglutide, in particular, has gained popularity due to its potent effects on weight loss and glycemic control. However, reports of inappropriate off-label use have raised concerns about rare but serious complications [12,13].

While pre-approval clinical trials rigorously evaluate the efficacy of GLP-1RAs, the sample sizes and durations of patient observation in these trials are often insufficient to detect rare AEs or long-term safety issues. Moreover, strict inclusion and exclusion criteria may limit the generalizability of trial findings to real-world patient populations. Thus, robust postmarket surveillance is necessary for monitoring drug safety in diverse patient populations over extended periods of time [14]. The FDA Adverse Event Reporting System (FAERS) is a publicly accessible centralized repository for reports of AEs associated with FDA-approved drugs and biologic products [15]. Most studies evaluating the risk of GLP-1RAs using FAERS data have examined their association with gastrointestinal [16-19] or psychiatric [20,21] AEs, most of which were classified as nonserious. However, the association of GLP-1RAs with mortality and serious AEs remains unclear. This study investigated the association of GLP-1RAs with mortality and serious AEs via a disproportionality analysis of events reported to the FAERS database.

## Materials and methods

Study design

We conducted a disproportionality analysis to investigate AE reporting patterns associated with FDA-approved GLP-1RAs. This retrospective study examined AE reports submitted to the FAERS for all GLP-1RAs approved between Q2 2005 (coinciding with the initial FDA approval of exenatide (Byetta)) and Q1 2024. Institutional review board approval was not required because this study utilized de-identified data from a public repository per the US Department of Health and Human Services Policy for Protection of Human Research Subjects (45 CFR 46.101(b)(4)).

Data source

The FAERS database is a postmarket surveillance tool maintained by the FDA to monitor the real-world safety profiles of approved drugs and biologics. Healthcare professionals, consumers, and manufacturers voluntarily submit AE reports, medication error reports, and product quality complaints resulting in AEs to this centralized repository. The structure of FAERS adheres to the international safety reporting guidance (ICH E2B) issued by the International Council for Harmonisation of Technical Requirements for Pharmaceuticals for Human Use to ensure standardized data collection and facilitate global pharmacovigilance efforts. Reported AEs and medication errors are coded using the Medical Dictionary for Regulatory Activities to maintain consistency and enable analysis of AEs across different products and therapeutic areas. Clinical reviewers at the FDA continuously evaluate these reports to identify potential safety signals that may warrant further investigation or regulatory action, such as labeling changes, risk management plans, or marketing withdrawal.

GLP-1RA selection

We identified all GLP-1RAs approved by the FDA through March 31, 2024, for treating overweight/obesity or type 2 diabetes mellitus. The GLP-1RAs included in this study, in order of FDA approval date, were Byetta (AstraZeneca), Victoza (Novo Nordisk), Trulicity (Eli Lilly), Saxenda (Novo Nordisk), Bydureon (AstraZeneca), Bydureon BCise (AstraZeneca), Ozempic (Novo Nordisk), Rybelsus (Novo Nordisk), Wegovy (Novo Nordisk), Mounjaro (Eli Lilly), and Zepbound (Eli Lilly) [22]. We excluded fixed-ratio GLP-1RA/insulin combination drugs (i.e., Xultophy and Soliqua) due to the potential confounding of their AE profiles by the insulin component. GLP-1RAs with fewer than 100 reported AEs in FAERS (i.e., Adlyxin) were also omitted, as this limited sample size may not provide meaningful safety signal detection (Table 1).

**Table 1:** FDA-approved GLP-1RAs * Excludes GLP-1RA/insulin combination drugs (i.e., Xultophy (liraglutide + insulin degludec) and Soliqua (lixisenatide + insulin glargine)) and drugs with fewer than 100 total cases (i.e., Adlyxin (lixisenatide)) ** Dual gastric inhibitory polypeptide receptor and GLP-1RA. AE, adverse event; BID, twice a day; GLP-1RA, glucagon-like peptide-1 receptor agonist; QD, once a day; QW, once a week

Generic name	Brand name	Indication	FDA approval year	Dosing	Half-life (hours)	Total AEs	Mortality AEs	Serious AEs
Exenatide	Byetta	Type 2 diabetes mellitus	2005	Injection; BID	2	48,898	1,571 (3.2%)	12,423 (25.4%)
Liraglutide	Victoza	Type 2 diabetes mellitus	2010	Injection; QD	13	30,076	1,016 (3.4%)	11,166 (37.1%)
Dulaglutide	Trulicity	Type 2 diabetes mellitus	2014	Injection; QW	120	65,038	942 (1.4%)	12,116 (18.6%)
Liraglutide	Saxenda	Obesity/overweight	2014	Injection; QD	13	5,496	62 (1.1%)	2,221 (40.4%)
Exenatide	Bydureon (BCise)	Type 2 diabetes mellitus	2017	Injection; QW	168-336	29,824	353 (1.2%)	4,890 (16.4%)
Semaglutide	Ozempic	Type 2 diabetes mellitus	2017	Injection; QW	168	20,587	252 (1.2%)	9,031 (43.9%)
Semaglutide	Rybelsus	Type 2 diabetes mellitus	2019	Oral; QD	168	3,310	66 (2.0%)	1,417 (42.8%)
Semaglutide	Wegovy	Obesity/overweight	2021	Injection; QW	168	4,129	34 (0.8%)	1,194 (28.9%)
Tirzepatide**	Mounjaro	Type 2 diabetes mellitus	2022	Injection; QW	120	30,283	110 (0.4%)	2,609 (8.6%)
Tirzepatide**	Zepbound	Obesity/overweight	2023	Injection; QW	120	1,560	0 (0.0%)	49 (3.1%)

Outcomes

We evaluated all AE reports submitted to FAERS through March 31, 2024, in which a GLP-1RA was indicated as the primary suspect medication. We removed duplicate reports with identical primary identification and case numbers to avoid overestimating AE frequencies. FDA evaluators link mortality data to one or more AEs based on a review of all information provided in each case report, including the narrative description, reported cause of death, and temporal relationship between the AE and the fatal outcome. These evaluators then classify the seriousness of AEs according to predefined criteria outlined in the Code of Federal Regulations (21 CFR 314.80). Serious AEs encompass those associated with death, life-threatening events, inpatient hospitalization or prolongation of existing hospitalization, persistent or significant disability/incapacity, congenital anomaly/birth defects, or situations requiring medical intervention to prevent permanent impairment or damage.

Statistical analysis

The Cochran-Armitage trend test was performed to assess the association between the order of FDA approval for GLP-1RAs and the age and sex distribution of patients experiencing serious AEs. We calculated the reporting odds ratio (ROR) for mortality and serious AEs for each GLP-1RA compared to the combined group of all other GLP-1RAs. The ROR represents the ratio of the odds of reporting a complication for a particular medication relative to the odds of reporting the same complication for the remaining medications in the same therapeutic class [23]. An ROR greater than 1.0 indicates higher odds of the complication being reported for the drug of interest compared to the class comparator. A signal of disproportionate reporting for a GLP-1RA was identified if the lower bound of the 95% CI for the ROR exceeded 1.0 [23].

## Results

**Figure 1:**
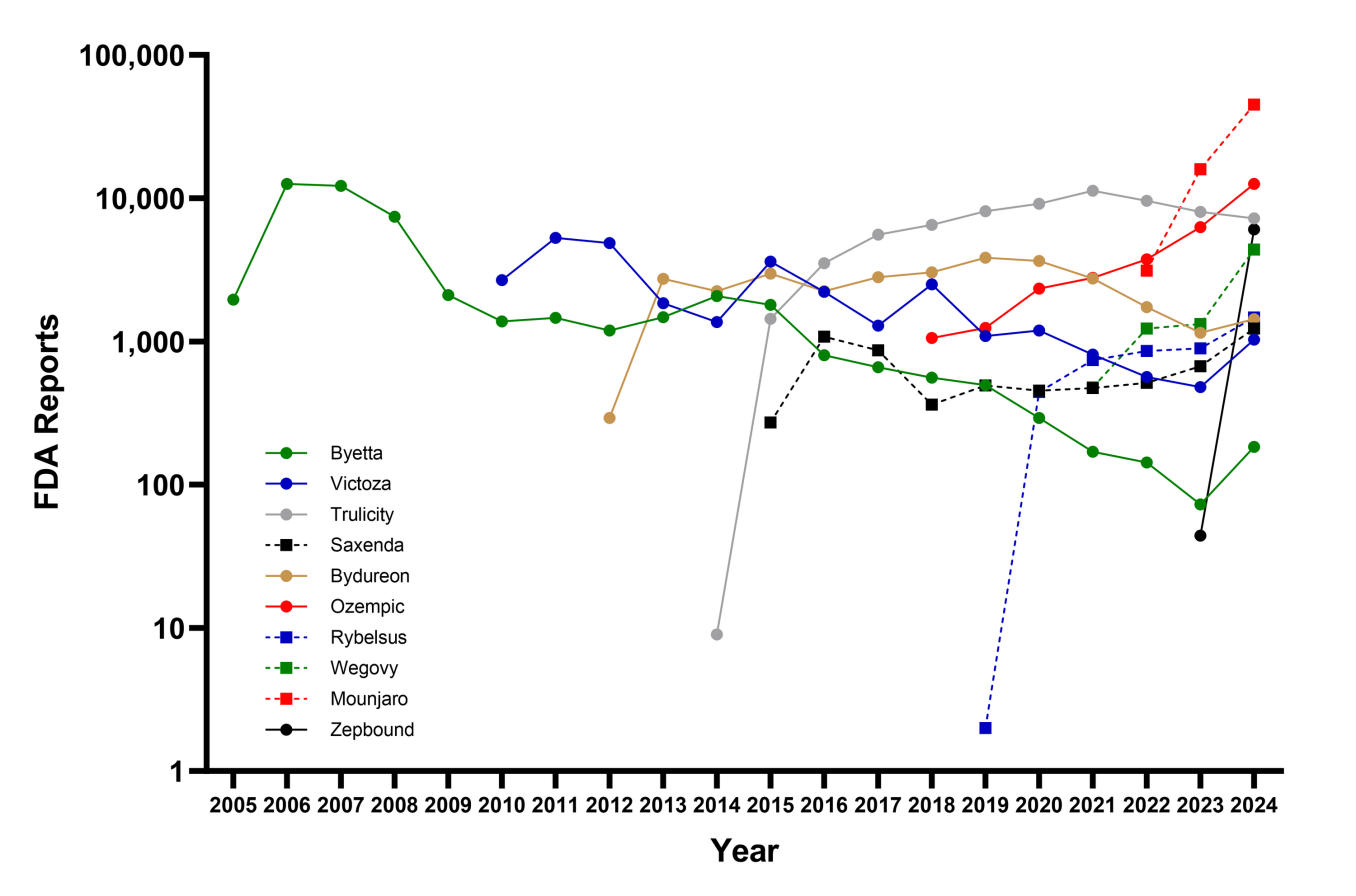
Annual trends in AE reports for GLP-1RAs in the FAERS database (2005-2024) Data are reported through March 31, 2024. For the year 2024, the data have been annualized to represent the full year based on the information available. AE, adverse event; FAERS, FDA Adverse Event Reporting System; GLP-1RA, glucagon-like peptide-1 receptor agonist

After filtering duplicate reports, our analysis included 287,201 unique AE reports through March 31, 2024, associated with FDA-approved GLP-1RAs that had at least 100 reported AEs in the FAERS database. The drugs with the highest total number of AE reports were Trulicity (65,038), Byetta (48,898), and Mounjaro (30,283) (Figure 1). The GLP-1RAs with the highest percentage of AE reports associated with mortality were Victoza (3.4%), Byetta (3.2%), and Rybelsus (2.0%), and those with the highest percentage of serious AEs were Ozempic (43.9%), Rybelsus (42.8%), Saxenda (40.4%), and Victoza (37.1%).

The mean age of patients experiencing serious AEs ranged from 47 years for Saxenda to 63 years for Bydureon (BCise). The majority of patients experiencing serious AEs were female for each drug, in particular for Saxenda (81%), Wegovy (78%), and Zepbound (77%). The newer GLP-1RAs were associated with a higher proportion of serious AEs reported in younger patients (p < 0.001) and females (p < 0.001). Consumer-reported serious AEs were most common for Zepbound (92%) and Mounjaro (85%). There was also a higher proportion of serious AEs reported in the US for the newer tirzapatide drugs (Table 2).

**Table 2:** Serious AE reporting with FDA-approved GLP-1RAs AE, adverse event; GLP-1RA, glucagon-like peptide-1 receptor agonist

Generic name	Brand name	Mean age (year)	Female sex	Reported by consumers	Reported in the United States
Exenatide	Byetta	60 (11)	58%	62%	58%
Liraglutide	Victoza	59 (12)	56%	35%	50%
Dulaglutide	Trulicity	62 (13)	52%	63%	66%
Liraglutide	Saxenda	47 (13)	81%	44%	41%
Exenatide	Bydureon (BCise)	63 (11)	54%	55%	67%
Semaglutide	Ozempic	59 (14)	60%	51%	58%
Semaglutide	Rybelsus	62 (14)	52%	26%	49%
Semaglutide	Wegovy	48 (13)	78%	47%	58%
Tirzepatide**	Mounjaro	56 (14)	65%	85%	76%
Tirzepatide**	Zepbound	49 (12)	77%	92%	67%
Overall		59 (13)	58%	54%	59%

**Figure 2:**
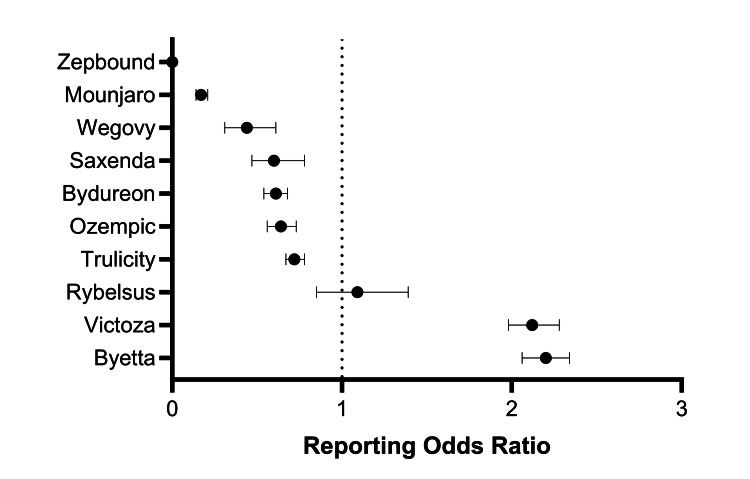
Forest plot showing the association between mortality and GLP-1RAs based on data in the FAERS database The ROR represents the odds of mortality with the drug of interest compared to the odds of mortality occurring among all other GLP-1RAs. An ROR greater than 1.0 indicates that the drug had greater odds of reporting mortality relative to the other drug comparators. A significant safety signal is represented by an ROR with a 95% CI lower bound greater than 1.0. FAERS, FDA Adverse Event Reporting System; GLP-1RA, glucagon-like peptide-1 receptor agonist; ROR, reporting odds ratio

Disproportionality analyses revealed significant safety signals for reports of mortality and serious AEs associated with certain GLP-1RAs. Compared to the composite of other drugs in this class, the earliest approved GLP-1RAs, Byetta and Victoza, demonstrated higher odds of mortality, with RORs of 2.20 (95% CI: 2.06-2.34) and 2.12 (95% CI: 1.98-2.28), respectively. In contrast, Trulicity, Saxenda, Wegovy, and Mounjaro had lower RORs of mortality, with all 95% CIs less than 1.0 (Figure 2).

**Figure 3:**
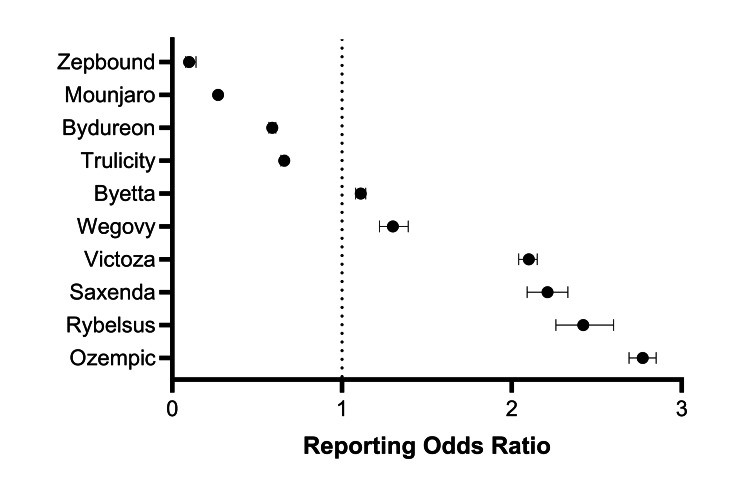
Forest plot showing the association between serious AEs and GLP-1RAs based on data in the FAERS database The ROR represents the odds of a serious AE with the drug of interest compared to the odds of a serious AE occurring among all other GLP-1RAs. An ROR greater than 1.0 indicates that the drug had greater odds of reporting a serious AE relative to the other drug comparators. A significant safety signal is represented by an ROR with a 95% CI lower bound greater than 1.0. AE, adverse event; FAERS, FDA Adverse Event Reporting System; GLP-1RA, glucagon-like peptide-1 receptor agonist; ROR, reporting odds ratio

For serious AEs, the ROR signals were elevated for the semaglutide products Ozempic (2.77, 95% CI: 2.69-2.85), Rybelsus (2.42, 95% CI: 2.26-2.60), and Wegovy (1.30, 95% CI: 1.22-1.39); the liraglutide products Victoza (2.10, 95% CI: 2.04-2.15) and Saxenda (2.21, 95% CI: 2.09-2.33); and Byetta (1.11, 95% CI: 1.08-1.14) compared to the other GLP-1RAs. Conversely, Trulicity, Bydureon, Mounjaro, and Zepbound had lower RORs for serious AEs, with all 95% CIs less than 1.0 (Figure 3).

## Discussion

Disproportionality analysis is an important pharmacovigilance tool for the early detection of rare but potentially serious AEs that may not be fully appreciated during the pre-marketing phase of drug development [24]. The present analysis of the FAERS database revealed notable differences in disproportionality signals for mortality and serious AEs among the various GLP-1RAs approved for the treatment of overweight/obesity and type 2 diabetes. The earliest approved GLP-1RAs, Byetta and Victoza, demonstrated significantly elevated RORs for mortality compared to other GLP-1RAs. In contrast, Trulicity, Saxenda, Wegovy, and Mounjaro had lower mortality reporting signals. For serious AEs, elevated RORs were seen with Victoza, Saxenda, Ozempic, Rybelsus, Wegovy, and Byetta, while Trulicity, Bydureon, Mounjaro, and Zepbound exhibited lower serious AE reporting odds. These variations in real-world safety reporting profiles of GLP-1RAs highlight the importance of individually evaluating drugs within the same pharmacological class since they may not be entirely interchangeable from a patient risk perspective.

The results showed that the proportion of female patients reporting serious AEs with GLP-1RAs has been increasing, while the age of affected patients has been decreasing over time. While 62% of patients receiving GLP-1RAs are female [10], the proportion of females reporting SAEs in FAERS was markedly higher with Saxenda (81%), Wegovy (78%), and Zepbound (77%). Thus, our findings raise questions about potential differential risks by sex that should be explored further. As the demographic of patients reporting SAEs shifts toward a younger and more female-dominant population [10], it will be important to identify the unique physiological, psychosocial, and behavioral factors that may influence the risk of adverse outcomes in these groups. Similarly, it is important to examine these risks among various racial and socioeconomic classes, as lower rates of GLP-1RA use have been reported among Asian, Black, and Hispanic individuals and those with lower household incomes [25]. Due to potential disparities in healthcare access and utilization and the absence of these data in the FAERS database, there may be less data available for these populations, underscoring the need to fully characterize the risks associated with GLP-1RAs.

The higher proportion of consumer-reported events for Zepbound and Mounjaro, the most recently approved GLP1-RAs, may indicate a growing patient awareness and willingness to monitor and report their experiences with these newer agents. This increased patient involvement in pharmacovigilance is a trend that should be encouraged and supported by healthcare providers and regulatory agencies. By leveraging patient-reported outcomes and experiences, a more comprehensive understanding of the real-world safety and effectiveness of these medications may be obtained. Additionally, as patients become more active participants in their care and the postmarket surveillance of medications, the quality and completeness of AE reports may improve [26]. This, in turn, may enhance the ability of regulatory authorities and healthcare professionals to promptly identify and respond to emerging safety signals.

The findings of the current study highlight the importance of carefully weighing the potential risks of GLP-1RAs against their well-established benefits in promoting weight loss in overweight or obese patients and improving glycemic control in individuals with type 2 diabetes mellitus. While the safety signals identified in this analysis warrant attention and further investigation, they should be considered in the context of the substantial cardiometabolic benefits offered by these agents. Numerous studies have demonstrated the ability of GLP-1RAs to reduce the risk of major adverse cardiovascular events, improve glucose homeostasis, enhance insulin sensitivity, and promote weight loss in overweight/obese individuals [8,9]. It is important to recognize that while the AE profile of GLP-1RAs is an important driver of patient preference, other attributes such as drug efficacy, dosing frequency, and administration method also significantly contribute to shaping patient treatment preferences [27]. Therefore, a comprehensive approach to treatment decision-making should consider all factors influencing a patient’s willingness and ability to adhere to GLP-1RA therapy.

The present study has several important limitations to consider when interpreting the findings. First, the FAERS database relies on voluntary reporting of AEs by healthcare professionals, patients, and manufacturers. This voluntary reporting system is susceptible to underreporting and reporting bias. In fact, it has been estimated that only 20-33% of serious AEs are reported to FAERS [28], suggesting that most serious AEs associated with GLP-1RAs likely remain unreported. Second, the reports in the FAERS database do not establish a causal relationship between the suspected drug and the reported AEs since they may be related to the underlying disease being treated, other concomitant medications, or other unrelated factors. Furthermore, many reports contain multiple AEs, making it challenging to determine which specific event(s) directly contributed to the death or serious AE. Due to these limitations, the present study did not analyze individual AEs, as drawing conclusions about causality would be inappropriate. Third, FAERS contains limited information on variables like dosing, duration of treatment, patient demographics, and comorbidities. The absence of these details in the FAERS database hinders the ability to identify potential risk factors and fully characterize the safety profile of the GLP-1RAs studied. Fourth, this study could not account for changes in reporting rates over time, evolving prescribing practices, or off-label use of the drugs. These factors may introduce variability in the data and make it challenging to compare results across different time periods or between drugs. Finally, the findings of this study may not be generalizable to GLP-1RAs currently in development or those used in countries outside the US. The safety profile of newer agents or those used in different healthcare systems may differ from the drugs included in this analysis, and caution should be exercised when extrapolating these results to other settings. Despite these limitations, the present study highlights the need for ongoing postmarket surveillance and further research to better characterize the risks associated with GLP-1RAs in clinical practice.

## Conclusions

This pharmacovigilance study utilizing the FAERS database identified potential safety signals of increased mortality and serious AE reporting associated with certain GLP-1RAs, particularly the earlier approved liraglutide agents Byetta and Victoza. While these results do not definitively establish causality, these findings highlight the importance of proactive postmarket surveillance to characterize the real-world safety profiles of individual GLP-1RAs. Future studies should explore the underlying mechanisms of the observed safety signals and identify patient subgroups that may be at higher risk of experiencing adverse outcomes with specific GLP-1RAs. Such research would inform more personalized treatment approaches.
